# Exploring the Leaves of *Annona muricata* L. as a Source of Potential Anti-inflammatory and Anticancer Agents

**DOI:** 10.3389/fphar.2018.00661

**Published:** 2018-06-20

**Authors:** Siti Mariam Abdul Wahab, Ibrahim Jantan, Md. Areeful Haque, Laiba Arshad

**Affiliations:** Drug and Herbal Research Center, Faculty of Pharmacy, Universiti Kebangsaan Malaysia, Kuala Lumpur, Malaysia

**Keywords:** *Annona muricata* leaves, phytochemistry, anti-inflammatory effects, anticancer effects, neurotoxic acetogenins

## Abstract

The use of anti-inflammatory natural products to treat inflammatory disorders for cancer prevention and therapy is an appealing area of interest in the last decades. *Annona muricata* L. is one of the many plant extracts that have been explored owing to their anti-inflammatory and anticancer effects. Different parts of *A. muricata* especially the leaves have been used for various ethnomedicinal purposes by traditional healers to treat several diseases including cancer, inflammation, diabetes, liver diseases, and abscesses. Some of these experience-based claims on the use of the plant have been transformed into evidence-based information by scientific investigations. The leaves of the plant have been extensively investigated for its diverse pharmacological aspects and found eminent for anti-inflammatory and anticancer properties. However, most studies were not on the bioactive isolates which were responsible for the activities but were based on crude extracts of the plant. In this comprehensive review, all significant findings from previous investigations till date on the leaves of *A. muricata*, specifically on their anti-inflammatory and anticancer activities have been compiled. The toxicology of the plant which has been shown to be due to the presence of neurotoxic annaceous acetogenins and benzyltetrahydro-isoquinoline alkaloids has also been updated to provide recent information on its safety aspects. The present knowledge of the plant has been critically assessed, aimed at providing direction toward improving its prospect as a source of potential anti-inflammatory and anticancer agents. The analysis will provide a new path for ensuring research on this plant to discover new agents to treat inflammatory diseases and cancer. Further *in vitro* and *in vivo* studies should be carried out to explore the molecular mechanisms underlying their anti-inflammatory responses in relation to anticancer activity and more detail toxicity study to ensure they are safe for human consumption. Sufficient preclinical data and safety data generated will allow clinical trials to be pursued on this plant and its bioactive compounds.

## Introduction

Inflammation, owing to central element of innate immunity and inflammatory response serve as a protective mechanism emerged in higher animals in order to defend them against injury and infection. In reaction to any tissue damage, a multifactorial network of chemical signals is initiated in the human body which keep the host body response and repair the impaired tissue. This multifactorial network encompasses activation and migration of different inflammatory cells i.e., monocytes, neutrophils, and eosinophils from the venous system to sites of injury. The coordination of the recruitment of aforementioned inflammatory cells to sites of tissue injury and to the provisional extracellular matrix (ECM) involves a four-step mechanism. The first step is the activation of adhesion molecules such as L-, P-, and E-selectin, expedite rolling along the vascular endothelium. Next step is the triggering of signals mediated by leukocyte-activating molecules and cytokines leading to activation and upregulation of leukocyte integrins. Thirdly, neutrophils are immobilized on the surface of the vascular endothelium by tight adhesion through α_4_β_1_ and α_4_β_7_ integrins further binding to endothelial vascular cell-adhesion molecule-1 (VCAM-1) and MadCAM-1, respectively. Lastly, the transmigration of the cells to the site of injury through the endothelium is supposedly mediated by extracellular proteases, such as matrix metalloproteinases (MMPs) (Chettibi et al., 1999). In the main, inflammation is segregated as acute inflammation and chronic inflammation. The acute inflammatory phase is characterized by increased in blood flow, aggregation of fluid, different cytokines and leukocytes and vascular permeability (Feghali and Wright, 1997). Nonetheless it resides for short time and is usually self-limiting because of the production of anti-inflammatory cytokines followed by pro-inflammatory cytokines ultimately resulting in tissue remodeling and normal tissue homeostasis. Notwithstanding, if there is unabating inflammation or if the system fails to return to normal homeostasis it eventually leads to chronic inflammation which is identified as a root cause in the development of a variety of chronic inflammatory and immune-related diseases (Arshad et al., 2017a). Chronic inflammation is correlated to the progression of human diseases such as arthritis, cancer, allergy, infectious diseases, arteriosclerosis, and autoimmune disorders (Medzhitov, 2008). Most of the immune system disorders are usually described by overactivity of the immune cells or abnormally low activity of the immune system. The body attacks and damages its own tissues, a condition known as acquired immune system reaction (autoimmune diseases) in cases of hyperactivity of immune system while immune deficiency diseases decrease the body's ability to fight invading pathogens, causing vulnerability to infections. Cancer, rheumatoid arthritis, Type 1 diabetes, systemic lupus erythematosus, tuberculosis and atherogenesis, are among the most common diseases due to suppression of the immune functions.

Immunoediting which describes the relation between immune response and tumor development occurs in three phases. During the elimination phase acute inflammatory response induced by innate and adaptive immune system occurs to recognize and eliminate the early-generated tumor cells by induction of apoptosis. Next, the tumor cells start to resist the strong immune surveillance and shift into the equilibrium phase between the tumor proliferation and apoptosis. In the escape phase, tumor cells become less immunogenic and able to evade the immune control (Mohamed et al., 2017). Chronic inflammation (e.g., chronic inflammatory bowel diseases, chronic gastritis, prostatitis, ulcerative colitis) may increase the incidence of cancer generation. Accumulation of bioactive cytokines, chemokines, ROS and growth factors by the immune cells will induce mutation development and transition of the normal cells into abnormal tumor cells (Balkwill, 2009). The proliferation of normal cells is enhanced during tissue injury associated with wounding, along with the regeneration of tissue, as the invading pathogen is removed, proliferation, and inflammation abated and the repair is completed. On the other hand, proliferating cells that endure DNA damage continue to proliferate in microenvironments and growth factors support their growth. Eventually, the tumor microenvironment is said to be largely concentrated with inflammatory cells, which is considered a crucial element in the neoplastic process, further stimulating proliferation, survival, and migration. Additionally, tumor cells are also assimilated with some of the signaling molecules of the innate immune system, such as chemokines, selectins, and their receptors for invasion, migration, and metastasis (Dvorak, 1986).

Natural products especially plant-derived compounds have been classified as a significant class of novel immunomodulators particularly as potent anti-inflammatory and anticancer agents. The anti-inflammatory and anticancer properties of plant-based therapeutics have attracted the interest of researchers as they provide alternative strategies to manage several infectious and debilitating diseases. A number of plant extracts have been suggested as potent immunomodulators with promising anti-inflammatory and anticancer properties such as *Annona muricata* L., *Catharanthus roseus* (L.) G. Don, *Camptotheca accuminata* Decne., *Centella asiatica* (L.) Urb., *Ipomoea batatas* (L.) Lam, *Phyllanthus amarus* Schumach. & Thonn*., Phyllanthus niruri* L., *Picrorhiza scrophulariiflora* Pennell*, Trigonella foenum graecum* L., and *Zingiber zerumbet* (L.) Roscoe ex Sm. (da Rocha et al., 2001; Jantan et al., 2015; Ahmad et al., 2016; Ilangkovan et al., 2016; Laksmitawati et al., 2016; Haque and Jantan, 2017; Haque et al., 2017a). Furthermore, phytochemicals such as alkaloids, terpenoids, polysaccharides, lactones, flavonoids, carotenoids, and glycosides as well as essential oils isolated from several plants have also been shown to exhibit potential anti-inflammatory and anticancer effects (Jantan et al., 2015; Arshad et al., 2017b; Haque and Jantan, 2017; Haque et al., 2017a,b; Mohamed et al., 2017). The plant extracts and their active constituents with anti-inflammatory potential serve as natural resources and may provide us with valuable entities to develop novel anti-inflammatory agents to be used as supplements or adjuvants with the present therapeutic modalities for chemotherapy. This review is to provide an updated overview of the potential anti-inflammatory and anticancer roles of the leaves of *Annona muricata* L., their underlying mechanisms of modulating the immune-associated molecular targets, and critically assessed its importance in providing new leads for the development of new anti-inflammatory and anticancer agents. In addition, phytochemistry, and toxicological information have also been included to correlate its efficacy and clarify its safety profile.

## Distribution and taxonomy

**Figure 1:**
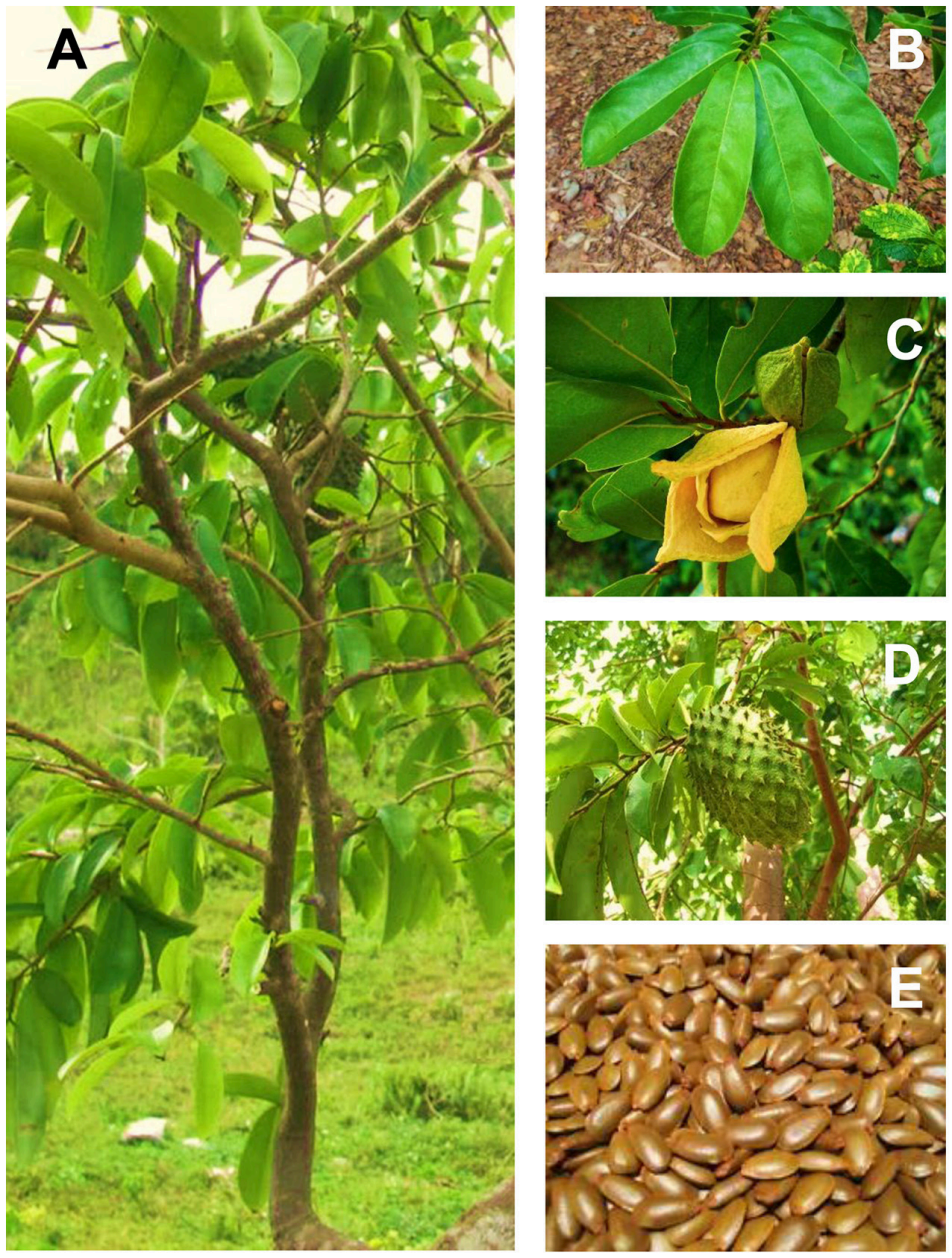
*Annona muricata* L. **(A)** Whole plant **(B)** Leaves **(C)** Flowers **(D)** Fruits **(E)** Seeds.

*Annona muricata* is widely known as soursop due to the sour and sweet taste of its fruit. It is also known as prickly custard apple due to its taste. The fruit is locally referred as durian belanda. In Indonesia, the plant is called sirsak or nangka belanda, while it is known as graviola in Portuguese and guanabana in Latin American. It is also recognized by other indigenous names such as annone, anona, graviola, araticum grande, araticum-manso, coronsol, corossol épineux, grand corossol, coração-de-rainha, guanábana, guanábano, gurúsulu, jaca-do-pará, kaoraosaly' jaca-de-pobre, cachiman épineux, mkononono, anoda, pumo, puntar waihia, quanabana, saput, sauersack, stachelannone, taggannona, and zuurzak. This plant has the taxonomic classification of the kingdom of Plantae, the division of Angiosperms (Magnoliophyta), the class of Magnolids, the order of Magnoliales, the family of Annonaceae, the genus of *Annona* and the species of *A. muricata* L. (Pinto et al., 2005a; Gavamukulya et al., 2017). The accepted full name of this species is *A. muricata* L. with other synonyms; *A. muricata* var*. borinquensis* Morales, *A. muricata f. mirabili*s R.E.Fr., *A. bonplandiana* Kunth, *A. cearaensis* Barb. Rodr., *A. macrocarpa* Wercklè and *Guanabanus muricatus* M. Gómez. The Annonaceae family consists of approximately 130 genera and 2300 species, while the genus *Annona* comprises over 70 species among which *A. muricata* is the most widely grown (Leboeuf et al., 1980; Mishra et al., 2013; Coria-Téllez et al., 2016). *A. muricata* is endemic to the warmest areas of the tropics of South and Central America and the Caribbean. It is now widely found in tropical and subtropical regions of Central and South America, Western Africa, and Southeast Asia at the altitudes below 1,200 m above sea level with temperature ranging from 25 to 28°C and relative humidity between 60 and 80% and have the annual rainfall above 1500 mm (Adewole and Caxton-Martins, 2006; Coria-Téllez et al., 2016). It is a tree 5–8 m in height, 15–83 cm in diameter and features an open, roundish canopy with large, glossy, dark green leaves (González et al., 2004). The tree tends to bloom and fruit most of the year, but there are more defined seasons depending on the altitude (Pinto et al., 2005a). Its fruits are edible, heart-shaped, green in color and 15–20 cm in diameter. The flesh is white and creamy with a characteristic aroma and flavor. Each fruit may contain 55-170 black seeds when fresh and they turn light brown when dry (Awan et al., 1980). Figure 1 refers the various plant parts of *A. muricata*. The full scientific names, synonyms, vernacular/ common names, classification, distributional range, and consumed parts of *A. muricata* are summarized in Table 1.

**Table 1:** General information of *Annona muricata* L.

**Species**	**Accepted full name**	**Synonyms**	**Taxonomy**	**Availability**	**Common/ vernacular name**	**Plant parts in use**	**References**
*Annona muricata*	*Annona muricata* L.	*Annona muricata var. Borinquensis* Morales; *Annona muricata f. mirabili*s R.E.Fr.; *Annona bonplandiana* Kunth; *Annona cearaensis* Barb. Rodr.; *Annona macrocarpa* Wercklè; *Guanabanus muricatus* M. Gómez	Kingdom: Plantae Division: Angiosperms (Magnoliophyta) Class: Magnolids Order: Magnoliales Family: Annonaceae Genus: *Annona* Species: *Annona muricata*	Tropical regions of Central and South America, Western Africa and Southest Asia.	English: soursop Indonesian: sirsak, nangka belanda Malay: durian belanda Portugese: graviola Latin American Spanish: guanabana Other Indigenous names: annone, anona, araticum grande,araticum-manso, cachiman épineux, coração-de-rainha, coronsol, corossol épineux, grand corossol, graviola, guanábana, guanábano, gurúsulu, jaca-de-pobre, jaca-do-pará, kaoraosaly', mkononono, prickly custard apple, anoda, pumo, puntar waihia, quanabana, saput, sauersack, soursop, stachelannone, taggannona, zuurzak	Leaf, bark, root, seed, fruit, flower, stem, pulp	Pinto et al., 2005a; Moghadamtousi et al., 2015b; Coria-Téllez et al., 2016

## Traditional values

Different parts of *A. muricata* are widely used in traditional medicine of many countries to cure various ailments and diseases. The natives of Malaysia apply the leaf juice of a mixture of *A. muricata, A. squamosa* L. and *Hibiscus rosa-sinensis* L. on the head to protect against fainting, and they also use the *A. muricata* leaves to treat cutaneous (external) and internal parasites (Ong and Norzalina, 1999). The leaves have also been used to treat cystitis, diabetes, headaches, and insomnia (Adewole and Caxton-Martins, 2006; de Souza et al., 2009; Mishra et al., 2013). The decoction of the leaves is applied topically for its anti-rheumatic and neuralgic effects, and to reduce abscesses (Adewole and Caxton-Martins, 2006; de Souza et al., 2009; Mishra et al., 2013). The leaves are used in the bath to cure skin diseases in the Caribbean islands, Indonesia as well as in the South Pacific countries (Longuefosse and Nossin, 1996; Boulogne et al., 2011). In Ecuador, Mauritius, and New Guinea, the application of the *A. muricata* leaves is local on the pain site (Tene et al., 2007; Rai et al., 2009; Sreekeesoon and Mahomoodally, 2014). Decoctions of *A. muricata* leaves are used as analgesics in Brazil, Martinique, Mexico and Nicaragua (Longuefosse and Nossin, 1996; Ross, 2010), while in Benin, the Caribbean, Cuba, and Mexico, it is used to reduce colds, flu, and asthma (Joyeux et al., 1995; Beyra et al., 2004; Kossouoh et al., 2007; Haiat and Bucay, 2009). The importance of *A. muricata* leaves to treat malaria is very crucial in tropical countries such as Cameroon, Togo, and Vietnam (Ross, 2010; Boyom et al., 2011; Tan et al., 2015). In Ghana, *A. muricata* and some other plants are decocted into a mixture and used in bath for feminine hygiene (Asase et al., 2012).

Table 2 enlists the traditional medicinal uses that have been reported for the leaves of *A. muricata*, together with the regions in which they are used as well as the mode of uses. Besides the wide array of the ethnopharmacological values of the leaves of *A. muricata*, the juicy flesh-fruit of the soursop is a remedy for rheumatism, arthritic pain, fever, neuralgia, heart, and liver diseases, diarrhea, dysentery, malaria, parasites, skin rashes, and worms as well as increasing breast milk after childbirth (de Lima and Alves, 2011; Hajdu and Hohmann, 2012; Moghadamtousi et al., 2015a). In India, the roots, bark and leaves of *A. muricata* are claimed to exhibit antiphlogistic and anthelmintic activities while the flowers and fruits of the plant are applied to treat catarrh (Watt and Breyer-Brandwijk, 1962; Adewole and Ojewole, 2009). The seeds are used as an anti-anthelmintic against external and internal worms and parasites. *A. muricata* is employed in tropical Africa as insecticidal and pesticidal agents besides being used for the treatment of coughs, pain and skin diseases (Moghadamtousi et al., 2015a). The leaves, seeds, unripe fruits and roots of *A. muricata* are traditionally used as biopesticides, bioinsecticides and topical insect repellents and showed significant effectiveness among other pests in Latin America. The aqueous extract of *A. muricata* is used to control lepidopteran larvae, aphids and thrips, among others (Brechelt, 2004; Leatemia and Isman, 2004; Isman and Akhtar, 2007). In addition, the leaves, bark, and roots of *A. muricata* have been used for its anti-inflammatory, hypotensive, sedative, hypoglycemic, smooth muscle relaxant, and antiplasmodic effects (Adewole and Ojewole, 2009; Mishra et al., 2013). Besides the traditional medicinal uses, *A. muricata* is also used in other fields, for instance, the fruits are widely used in the food industries in the making of syrups, beverages, candy, ice creams and shakes (Wu et al., 1995a; Jaramillo-Flores and Hernandez-Sanchez, 2000).

**Table 2:** Traditional uses of the leaves of *Annona muricata* L.

**Country/ regions**	**Uses**	**Mode of uses**	**References**
Benin	Insomnia, catarrh, febrifuge	Decoction/ oral	Kossouoh et al., 2007
Bolivia	Kidney disorders, hypertension	Decoction/ oral	Hajdu and Hohmann, 2012
Brazil	Snake bite	Macerate/ topical	Ross, 2010; Ritter et al., 2012
	Analgesic	Decoction/ oral	
	Arthritis pain, rheumatism, neuralgia, weight loss	Decoction/ oral	Cercato et al., 2015
Cameroon	Malaria, anthelmintic, parasites, antimicrobial, anticonvulsant, digestive	Decoction/ oral	Boyom et al., 2011; Tsabang et al., 2012
	Typhoid fever	Decoction/ oral	Roger et al., 2015
Caribbean	Chills, febrifuge, flu, indigestion, nervousness		Joyeux et al., 1995; Taylor, 2002; Boulogne et al., 2011
Columbia	Febrifuge, inflammation	Decoction/ oral	Betancur-Galvis et al., 1999
Cuba	Catarrh	Decoction in water or milk/ oral	Beyra et al., 2004
Dominican Republic	Respiratory condition, women in labor		Ross, 2010; Vandebroek et al., 2010
	Galactogogues	Infusion/ oral	
Ecuador	Rheumatism	Heated/ topical	Tene et al., 2007
Guyana	Convulsion	Infusion/ oral	Taylor, 2002; DeFilipps et al., 2004
Haiti	Flu, heart affectation parasite, pellagra, anxiety		de Lima and Alves, 2011
India	Suppurative, febrifuge	Decoction/ oral	
	Pain and pus from ulcers	Smeared in coconut oil/ topical	Dagar and Dagar, 1991
Indonesia	Insecticidal		Leatemia and Isman, 2004; Roosita et al., 2008; de Lima and Alves, 2011
	Dermatitis		Abdillah et al., 2015
Jamaica	Febrifuge, parasites, diarrhea, lactagogue, dewormer	Decoction/ oral	de Lima and Alves, 2011
Madagascar	Heart palpitation, malaria, liver maladies	Decoction	Novy, 1997
Malaysia	Lice	Crushed/ topical	de Lima and Alves, 2011
Martinique	Skin rashes, sedative thoracic pain, inflammation	Crushed/ bath	Longuefosse and Nossin, 1996
	Flatulence, liver disease	Decoction/ oral	
Mauritius	Hypertension	Infusion /oral	Mootoosamy and Mahomoodally, 2014
	headache	Crushed/ topical	Sreekeesoon and Mahomoodally, 2014
Mexico	Gastric cancer, gastrointestinal disorders, stomach pain	Decoction/ oral	Alonso-Castro et al., 2011
	Febrifuge, diarrhea, dysentery, stomach pain	Infusion/ oral	Yasunaka et al., 2005
	Bronchitis, asthma, leprae	Infusion/ oral	Haiat and Bucay, 2009
Nicaragua	Ringworm	Plaster/ topical	Coe, 2008; Ross, 2010; Benavides González, 2011
	Abdominal and back pain, menstrual hemorrhage, abortions, fever, vaginal infection	Infusion/ oral	
	Renal and skin disorders, diarrhea	Decoction/ oral	
Nigeria	Gastric disorders, prostate cancer, diabetes, neuralgia	Decoction/ oral	Pinto et al., 2005b; Atawodi, 2011
Panama	Dyspepsia, allergy, helminthiasis		Gupta et al., 1979; Ross, 2010
Philippines	Lice, dandruff		Langenberger et al., 2009; de Lima and Alves, 2011; Ong and Kim, 2014
	Cancer, ascariasis, high blood pressure, stomach acidity, urination difficulty, cough, headache,	Decoction/ oral	
New Guinea	Stomach pain	Heated/ compression	Rai et al., 2009; de Lima and Alves, 2011
Peru	Obesity, gastritis, dyspepsia, diabetes, inflammation, cancer, spams, sedative, flu, febrifuge, anxiety, kidneys, prostate, urinary tract, infection, inflammation, panacea	Infusion/ oral	Bussmann et al., 2010; de Lima and Alves, 2011; Poma et al., 2011; Rodríguez, 2011; Monigatti et al., 2013
South pacific countries	Stomach ailments, indigestion, skin diseases	Infusion/ oral	Sotheeswaran et al., 1998
	Dizziness, fainting spells	Bath	Ross, 2010
Trinidad and Tobago	Hypertension		Lans, 2006; de Lima and Alves, 2011
Togo	Hypertension/ diabetes	Decoction/ oral	Ross, 2010; Karou et al., 2011
Uganda	Diabetes	Infusion/ oral	Ssenyange et al., 2015
Vanuatu	Scabies	Infusion/ bath	Bradacs et al., 2011
Venezuela	Liver affection, stomach pain, insecticidal	Decoction/ oral	Taylor, 2002; de Lima and Alves, 2011
West Africa	Sedative, nasopharyngeal affectation	Decoction/ oral	Burkill, 1985
West Indies	Asthma, diarrhea, hypertension, parasites, lactagogue, skin ailments	Decoction/ oral	Feng et al., 1962; Taylor, 2002; Ross, 2010; Boulogne et al., 2011
South Vietnam	Malaria	Infusion/ oral	Nguyen-Pouplin et al., 2007

## Phytochemistry

Phytochemical studies have been extensively carried out on different parts of *A. muricata* and till date, 212 secondary metabolites have been isolated and identified, such as acetogenins, alkaloids, phenolic compounds, and megastigmanes (Leboeuf et al., 1980; Yang et al., 2015; Coria-Téllez et al., 2016). *A. muricata* leaves have been reported to be a rich source of annonaceous acetogenins, a unique group of derivatives of long chain fatty acids derived from the polyketide pathway that belong to the family of Annonaceae (Sun et al., 2016). Thorough investigations have been carried out on the leaves of *A. muricata* as the leaves are the most utilized parts used for a wide array of ethnomedicinal uses. Acetogenins are the most predominant bioactive compounds of Annonaceace family, as well as of *A. muricata*. In the previous phytochemical investigations, more than 120 acetogenins have been reported from the leaves, stems, bark, seeds, pulp, and fruit peel of *A. muricata*, and, to date, approximately 46 acetogenins have been identified from the leaves. Wu and co-workers have isolated six annonaceous acetogenins from 95% ethanol extract of the leaves of *A. muricata* by subjecting the active fraction of the extract to repeated flash chromatography and high performance liquid chromatography (HPLC) to yield isolates namely, annomuricin A, annomuricin B, annomuricin C, muricatocin A, muricatocin B, and muricatocin C (Wu et al., 1995a,b,c). Zeng et al. (1996) isolated annopentocin A, annopentocin B, annopentocin C, *cis-*annomuricin-D-ones and *trans-*annomuricin-D-ones from the leaves of *A. muricata* (Zeng et al., 1996). Annonacin, annonacin A, annonacin B, annonacin-10-one, muricatalicin and muricatalin have been isolated and identified by Yu et al. (1997). Further investigation on the *A. muricata* leaf extract yielded annonaceous acetogenins, spectroscopically identified as annomuricine, muricoreacin, murihexocin C, and muricapentocin (Kim et al., 1998a,b). Further studies led to the isolation of annonacin, annonacinone, annocatalin, *cis*-corossolone, solamin, and corossolone (Liaw et al., 2002). Recent investigation conducted by Moghadamtousi et al. (2015c) on the ethyl acetate extract of *A. muricata* leaves afforded annomuricin E by using column chromatography approach. Notably, acetogenin is a long aliphatic chain of 35–38 carbons bonded to a γ-lactone α ring and terminally substituted by β-unsaturated methyl, in some cases, it is a ketolactone, with one or two tetrahydrofurans (THF) located along the hydrocarbons chain and a determined number of oxygen groups (hydroxyl, acetoxyls, ketones, epoxy). The previously identified acetogenins in *A. muricata* are mainly of mono-THF ring, although acetogenins have also been reported with two adjacent or nonadjacent THF rings. Moreover, acetogenins are linear and may have one or two epoxy groups (Coria-Téllez et al., 2016). Annonacin was the most abundant acetogenin isolated from the leaves of *A. muricata* (Champy et al., 2009).

Alkaloids are a diverse group of naturally occurring secondary compounds derived from amino acids or the process of transamination (Dey et al., 2017). Till date, around 22 alkaloids have been reported from *A. muricata* leaves. Anomurine and anomuricine, the isoquinoleic alkaloids were isolated and reported by Leboeuf et al. (1980). The chloroform extraction of *A. muricata* leaves yielded a crude extract with 0.125% of total alkaloids, which was later separated into nonphenolic and phenolic parts and subjected to column chromatography over silica gel. The nonphenolic fraction afforded aporphine alkaloids identified as anonaine, isolaureline, and xylopine, while the phenolic fraction led to the isolation of coclaurine, the benzyltetrahydroisoquinoline alkaloid (Fofana et al., 2011). Fofana et al. (2011) conducted further investigation on the alkaloid content of *A. muricata* leaves using chromatography over a column of silica gel. The alkaloids were eluted by various ratios of benzene and ethanol solvent system 99:1, 98:2, 95:5, and 90:10, yielded *N*-methylcoclaurine, asimilobin, remerine, isoboldine, and liriodenine. Subsequently, Matsushige et al. (2012b) isolated annonamine, (R)-O,O-dimethylcoclaurine, (S)-norcorydine and (R)-4′-O-methylcoclaurine. Based on previous investigations, reticuline and cureximine are the most abundant alkaloids in *A. muricata* (Leboeuf et al., 1982), and the leaves contained the highest alkaloid concentration compared to the roots, stems and fruits (Leboeuf et al., 1982; Fofana et al., 2011, 2012). Notably, isoquinoline, aporphine, and protoberberine typed-alkaloids were the most frequently isolated from the *A. muricata* (Mohanty et al., 2008).

Phenolic compounds are considered as the most important phytochemicals as most of them are soluble in water, since the most commonly used extract in traditional medicine is aqueous infusion (Coria-Téllez et al., 2016). Thirty-four phenolic compounds have been isolated from the leaves of *A. muricata*. The purification of n-butanol leaf extract of *A. muricata* led to the isolation of robinetin, taxifolin (+), quercetin, apigenin-6-C-glucoside, gallic acid, and luteolin 3′7-di-O-glucoside (George et al., 2012). Further investigation by Nawwar et al. (2012) resulted in the isolation of the argentinine, catechine, chlorogenic acid, epicatechine, kaempferol, kaempferol 3-O-rutinoside, quercetin 3-O-glucoside, quercetin 3-O-neohesperidoside, quercetin 3-O-robinoside, quercetin 3-O-α-rhamnosyl-(1-6)-β-sophorside, quercetin 3-O-α-rhamnosyl and quercetin-O-rutinoside. Recently, an investigation on the methanol and aqueous leaf extracts of *A. muricata* yielded cinnamic acid, coumaric acid, daidzein, emodin, gallocatechin, genistein, glycitein, homooorientin, isoferulic acid, and vitexin (George et al., 2015). Alongside, 14 megastigmanes, namely, annoionol A, annoionol B, annoionol C, annoionoside, vomifoliol, roseoside, turpinionoside A, citroside A, blumenol C, (+)-epiloliolide, loliolide, (1S,2S,4R)-trans-2-hydroxy-1,8-cineole β-D-glucopyranoside, (Z)-3-hexenyl β-D-glucopyranoside and rutin have also been isolated and reported to be present in the leaves of *A. muricata* (Matsushige et al., 2012b). Besides all the above mentioned phytochemicals, vitamins, amides, and essential oils have also been identified from the leaves of *A. muricata*. To date, 80 essential oils constituted mainly of sesquiterpenes derivatives have been identified in the leaves of this promising plant. All of the secondary metabolites isolated from the leaves of the *A. muricata* are compiled and summarized in Table 3.

**Table 3:** Major secondary metabolites isolated from the leaves of *Annona muricata* L.

**No**.	**Chemical group**	**References**
**ACETOGENINS**		
1.	Annonacin	Wu et al., 1995a; Liaw et al., 2002; Nakanishi et al., 2003; Champy et al., 2009
2.	(2,4-cis)-10R-annonacin-A-one	Wu et al., 1995a
3.	(2,4-trans)-10R-annonacin-A-one	Wu et al., 1995a
4.	Annohexocin	Zeng et al., 1996
5.	Annonacinone	Liaw et al., 2002; Nakanishi et al., 2003
6.	Annomuricin A	Wu et al., 1995a
7.	Annomuricin A	Wu et al., 1995a
8.	Annomuricin C	Wu et al., 1995a; Zeng et al., 1996; Moghadamtousi et al., 2015b
9.	Annomuricin E	Zeng et al., 1996; Moghadamtousi et al., 2015b
10.	Cis-annomuricin-D-one	Zeng et al., 1996; Alali et al., 1999
11.	Trans-annomuricin-D-one	Zeng et al., 1996; Alali et al., 1999
12.	Annomutacin	Wu et al., 1995a
13	Annonacin A	Wu et al., 1995a; Zeng et al., 1996
14.	Annopentocin A	Zeng et al., 1996; Alali et al., 1999
15.	Annopentocin B	Zeng et al., 1996; Alali et al., 1999
16.	Annopentocin C	Zeng et al., 1996; Alali et al., 1999
17.	Annonacinone	Liaw et al., 2002; Nakanishi et al., 2003
18.	Annocatalin	Liaw et al., 2002
19.	Annocatacin B	Chang et al., 2003
20.	Asimicinone-9-oxo	Champy et al., 2009
21.	Corossolin	Nakanishi et al., 2003
22.	Corossolone	Liaw et al., 2002
23.	Cis-corossolone	Liaw et al., 2002; Nakanishi et al., 2003
24.	Gigantecin	Champy et al., 2009
25.	Gigantetronin	Wu et al., 1995a
26.	Isoannonacin	Fang et al., 1993
27.	(2,4-cis)-isoannonacin	Wu et al., 1995b
28.	(2,4-trans)-isoannonacin	Wu et al., 1995b
29.	Montanacin D	Champy et al., 2009
30.	Montanacin E	Champy et al., 2009
31.	Montanacin H	Champy et al., 2009
32.	Muricatalicin	Yu et al., 1997
33.	Muricatalin	Yu et al., 1997
34.	Muricapentocin	Kim et al., 1998b; Alali et al., 1999
35.	Muricatocin A	Wu et al., 1995b; Alali et al., 1999
36.	Muricatocin B	Wu et al., 1995b; Champy et al., 2009
37.	Muricatocin C	Wu et al., 1995b
38.	Muricin H	Liaw et al., 2002
39.	Muricin I	Liaw et al., 2002
40.	Muricoreacin A	Kim et al., 1998b; Alali et al., 1999
41.	Muricoreacin B	Alali et al., 1999
42.	Murihexocin A	Zeng et al., 1996; Champy et al., 2009
43.	Murihexocin B	Zeng et al., 1996
44.	Murihexocin C	Kim et al., 1998b
45.	Solamin	Liaw et al., 2002; Nakanishi et al., 2003
46.	Cis-solamin	Alali et al., 1999
47.	Cis-solamin A	Konno et al., 2008
**ALKALOIDS**		
48.	Anonaine	Fofana et al., 2011; Matsushige et al., 2012a
49.	Annonamine	Matsushige et al., 2012a
50.	Asimilobine	Fofana et al., 2011
51.	Casuarine	Mohanty et al., 2008
52.	Coclaurine	Leboeuf et al., 1980; Fofana et al., 2011
53.	Coreximine	Leboeuf et al., 1980
54.	DMDP (2,5-Dihydroxymethyl-3,4, dihydroxypyrrolidine)	Mohanty et al., 2008
55.	DMJ (Deoxymannojirimycin)	Mohanty et al., 2008
56.	DNJ (Deoxynomirmycin)	Mohanty et al., 2008
57.	(R)-O,O-dimethylcoclaurine	Matsushige et al., 2012a
58.	(R)-4′-O-methylcoclaurine	Matsushige et al., 2012a
59.	Isoboldine	Fofana et al., 2011
60.	Isolaureline	Fofana et al., 2011
61.	Liriodenine	Fofana et al., 2011
62.	N-methylcoclaurine	Fofana et al., 2011
63.	N-methylcoculaurine	Kotake et al., 2004
64.	(S)-norcorydine	Matsushige et al., 2012a
65.	Remerine	Fofana et al., 2011
66.	Reticuline	Leboeuf et al., 1981; Kotake et al., 2004
67.	Stepharine	Leboeuf et al., 1981
68.	Swainsonine	Mohanty et al., 2008
69.	Xylopine	Fofana et al., 2011
**PHENOLS**		
70.	Apigenin-6-C-glucoside	George et al., 2012
71.	Argentinine	Nawwar et al., 2012
72.	Caffeic acid	Jiménez et al., 2014
73.	Caffeoylquinic acid	Marques and Farah, 2009
74.	Catechine	Nawwar et al., 2012
75.	Chlorogenic acid	Nawwar et al., 2012
76.	Cinnamic acid	George et al., 2015
77.	Coumaric acid	George et al., 2015
78.	Dicaffeoylquinic acid	Marques and Farah, 2009
79.	Feruloylquinic acid	Marques and Farah, 2009
80.	Daidzein	George et al., 2015
81.	Emodin	George et al., 2015
82.	Epicatechine	Nawwar et al., 2012
83.	Gallic acid	George et al., 2012
84.	Gallocatechin	George et al., 2015
85.	Genistein	George et al., 2015
86.	Gentisic acid	Taylor, 2002
87.	Glycitein	George et al., 2015
88.	Homooorientin	George et al., 2015
89.	Isoferulic acid	George et al., 2015
90.	Kaempferol	Nawwar et al., 2012
91.	Kaempferol 3-O-rutinoside	Nawwar et al., 2012
92.	Luteolin 3′7-di-O-glucoside	George et al., 2012
93.	Quercetin	George et al., 2012
94.	Quercetin 3-O-glucoside	Nawwar et al., 2012
95.	Quercetin 3-O-neohesperidoside	Nawwar et al., 2012
96.	Quercetin 3-O-robinoside	Nawwar et al., 2012
97.	Quercetin-O-rutinoside	Nawwar et al., 2012
98.	Quercetin 3-O-α-rhamnosyl	Nawwar et al., 2012
99.	Quercetin 3-O-α-rhamnosyl-(1-6)-β-sophorside	Nawwar et al., 2012
100.	Robinetin	George et al., 2012
101.	Tangeretin	George et al., 2015
102.	Taxifolin (+)	George et al., 2012
103.	Vitexin	George et al., 2015
**MEGASTIGMANES**		
104.	Annoionol A	Matsushige et al., 2012a,b
105.	Annoionol B	Matsushige et al., 2012a,b
106.	Annoionol C	Matsushige et al., 2012a,b
107.	Annoionoside	Matsushige et al., 2012a,b
108.	Vomifoliol	Matsushige et al., 2012b
109.	Roseoside	Matsushige et al., 2012b
110.	Turpinionoside A	Matsushige et al., 2012b
111.	Citroside A	Matsushige et al., 2012b
112.	Blumenol C	Matsushige et al., 2012b
113.	(+)-epiloliolide	Matsushige et al., 2012b
114.	loliolide	Matsushige et al., 2012b
115.	(1S,2S,4R)-trans-2-hydroxy-1,8-cineole β-D-glucopyranoside	Matsushige et al., 2012b
116.	(Z)-3-hexenyl β-D-glucopyranoside	Matsushige et al., 2012b
117.	Rutin	Matsushige et al., 2012b

## Anti-inflammatory activity

The anti-inflammatory activity of *A. muricata* leaves has been reported by many studies as listed in Table 4. The *in vitro* study conducted by Laksmitawati et al. (2016) revealed that *A. muricata* leaf extract possessed anti-inflammatory activity as it inhibited the inflammatory mediators, TNF-α, IL-1β, IL-6 and nitric oxide (NO). *A. muricata* leaf extract exhibited a remarkable difference on the TNF-α level in LPS-induced RAW264.7 cell line as compared to the positive control (LPS-stimulated cells without the extract) and demonstrated a worthy inhibitory activity with 46.8% inhibition. The administration of 50 μg/mL of *A. muricata* leaf extract in RAW264.7 cells resulted in 264.69 pg/mL of the TNF-α level, which was found comparable with the negative control (normal cell) (Laksmitawati et al., 2016). *A. muricata* leaf extract also significantly inhibited IL-6 and NO levels in an inflammation-induced cell at respective concentrations of 50 and 75 μg/mL to 63.89% (219.13 pg/mL) and 70.67% (9.79 μM), respectively. The study demonstrated the potential of *A. muricata* leaf extract in treating inflammation. Likewise, Moghadamtousi et al. (2015b) reported that *A. muricata* leaf extract showed anti-inflammatory activity during their investigation on wound healing potential of the extract on rats with excisional wound. The immunohistochemical evaluation in this study indicated the anti-inflammatory activity of the extract by revealing the up-regulation of HSP70. The ethanol extract of *A. muricata* leaves exhibited notable anti-arthritic activities in complete Freund's adjuvant (CFA)-induced arthritis in rats as reported by Chan et al. (2010). *A. muricata* extract at all doses (3, 10, 30, and 100 mg/kg) significantly reduced the edema by 48.39, 66.67, 79.57, and 72.04%, respectively, while at higher doses (30 and 100 mg/kg) significantly suppressed TNF-α level by 42.81 and 51.82% and IL-1β level by 35.57 and 39.79%, respectively, on day 14 post-CFA injection. The effects of the extract at the higher doses in suppressing TNF-α were stronger than the effect of indomethacin (51.82%).

**Table 4:** Anti-inflammatory activity of *A. muricata* leaf extract.

**Extract/fractions**	**Subject**	**Dose**	**Key findings**	**References**
Ethanol	RAW 264.7	50 μg/mL	The extract resulted in low TNF-α level in RAW264.7 (264.69 pg/mL), IL-1β level (905.00 pg/mL) and IL-6 (219.13 pg/mL). At 75 μg/mL the extract exhibited low NO level (9.79 μM) compared to untreated cells.	Laksmitawati et al., 2016
Water	Male Swiss albino mice, 5 weeks old, 25-30 g	250, 500, and 1000 mg/kg	Inhibited edema of the carrageenan-induced edema mice up to 26.82 and 52.70% inhibition at 250 and 500 mg/kg, respectively, reduced TPA-induced edema by 56 and 78% at 2.5 and 5 mg per ear respectively, and reduced MPO overproduction by 92.5% at 5 mg/ear.	Quilez et al., 2015
Water	Mouse	1.5 mg/kg	The plant reduced edema induced in the mouse model up to 71.12%	Poma et al., 2011
Ethanol	Rats	400 mg/kg	The carrageenan-induced paw edema in rats reduced by 0.47 mL.	de Sousa et al., 2010
80% Ethanol	Mice	10 mg/kg	The reaction of mice exposed to the hot plate prolonged up to 53.92%.	Hamid et al., 2012
		100 mg/kg	The time spent licking on formalin-induce mice reduced by 47.36%	
		300 mg/kg	The abdominal writhes of mice induced by 0.6% acetic acid inhibited by 95.3%.	
Ethanol	Mice	400 mg/kg	The latency time in mice increased by 13.25 min.	de Sousa et al., 2010
			Formalin-induced nociception in mice inhibited by 45%	
			Acetic acid-induced writhing in mice inhibited by 41.41%	
Ethanol	Rats	3, 10, 30, and 100 mg/kg	The extract significantly suppressed the TNF-α and IL-1β at 100 mg/kg.	Chan et al., 2010

In an *in vivo* study, different doses of *A. muricata* extract were assayed in carrageenan-induced inflammation and tetradecanoylphorbol acetate (TPA)-induced edema in mice. Myeloperoxidase activity (MPO) in inflamed tissue, MPO released by A-23187-stimulated rat neutrophils and NO released by murine macrophages were also determined (Quilez et al., 2015). The leaf extract at 250 and 500 mg/kg exhibited a significant edema reduction by 26.82 and 52.70%, respectively, in the carrageenan-induced edema model after the first h of observation. The study further highlighted that a single administration by oral gavage of *A. muricata* leaf extract at the concentration of 500 mg/kg was reported to significantly reduce the paw edema, as effective as 50 mg/kg of the standard ibuprofen. On the other hand, for the tetradecanoylphorbol acetate (TPA)-induced edema test, 56 and 78% of the edema was remarkably and dose-dependently reduced at the concentrations of 2.5 and 5 mg/ear of *A. muricata* leaf extract, respectively. A topical administration of the leaf extract of *A. muricata* notably and dose-dependently suppressed the overproduction of MPO by 92.5% of inhibition at the dose of 5 mg/ear. MPO was also reduced in activated rat neutrophils at 200 μg/ml while NO production was also dose-dependently suppressed in LPS-stimulated murine macrophages. In a nutshell, these finding revealed an outstanding anti-inflammatory properties of the extract of *A. muricata* leaves and directly confirmed the traditional uses of this species in treating inflammation. Analogously, another investigation reported that 1.5 mg/kg of the *A. muricata* leaf extract reduced 71.12% of carrageenan-induced edema in a mouse model (de Sousa et al., 2010). They suggested that the anti-inflammatory action of the plant extract was related to inhibition of one or more signaling intracellular pathways involved with the mediators (e.g., histamine, serotonin, bradykinin, substance P, and a platelet activating factor and prostaglandin). Moreover, the ethanol extract of *A. muricata* leaves had shown significant antinociceptive activity in a concentration-dependent manner when subjected to acetic acid-induced abdominal writhing in mice, formalin test in rats and hot plate test in mice (Hamid et al., 2012). For acetic acid-induced (0.6% acetic acid) writhing test of mice, the extract showed antinociceptive effect by significantly inhibiting the number of abdominal writhes, the potent activity was observed at a dose of 100 mg/kg. For formalin test, the administration of the extract inhibited biphasic characteristic response in the early as well as the late phase at all aforementioned doses. For hot plate test performed in mice, the extract extended the reaction time of mice (licking the paw or jumping) exposed to the hot plate when compared with control group and significantly increased the reaction time at the concentration of 100 mg/kg.

Apart from the leaves of *A. muricata*, the fruit has also been reported to exhibit immunomodulatory and anti-inflammatory activities. The methanol extract of fruit peel *A. muricata* at 6.25 and 100 μg/mL significantly suppressed the leukocytes expression of CD18/11a and exhibited 58.43 % of phagocytosis of leukocytes stimulatory activity at 100 μg/mL, comparable to the negative control. The fruit peel extract of *A. muricata* has potential to be used to treat health problems related to the immune system as it could modulate the innate immune system (Harun et al., 2015). Furthermore, investigation on the effect of fruit juice of *A. muricata* on ischemia-reperfusion injury in rats also confirmed that *A. muricata* fruit extract exhibited *in vivo* anti-inflammatory activities (Mohammed and Abbas, 2016). The anti-inflammatory effects of lyophilized fruit extract of *A. muricata* in mice was investigated using the carrageenan-induced rat paw edema and xylene-induced ear edema tests (Ishola et al., 2014). The time-dependent increase in paw circumference induced by carrageenan was inhibited by *A. muricata* treatment which was comparatively similar to that of diclofenac treated, while at 50 or 100 mg/kg pretreatment the xylene-induced ear edema was significantly reduced. The extract concentration-dependently inhibited both cyclooxygenase (COX)-1 and COX-2 activity by 39.44 and 55.71%, respectively, at 100 μg/mL.

On the contrary, Kim et al. (2016) reported the immunomostimulatory effect of standardized *A. muricata* leaf extract via activation of mitogen-activated protein kinase (MAPK) pathway in RAW 264.7 macrophages. The steam and ethanol extracts of *A. muricata* stimulated the mRNA expression of cytokines, including the pro-inflammatory cytokines, IL-1α and TNF-α, however only the steam extract up regulated inducible nitric oxide synthase (iNOS). The extracts also enhanced the production of TNF-α and nitrite, in consistence with their transcriptional expression. The authors suggested that *A. muricata* has the potential to be developed into an immune-boosting product for immuno-compromised patients. It is interesting to note that the results of this study contradicted the results of all previous studies which reported that the leaf extract of *A. muricata* possessed anti-inflammatory activity as it inhibited the inflammatory mediators. The contradicting results may be due to the use of different solvents and extraction procedures or the different chemical composition of the extracts used in the different studies. Further detailed mechanistic studies are required to ascertain the modulatory effects of the standardized *A. muricata* leaf extract on the immune system particularly on the pro-inflammatory mediators.

## Anticancer activity

### *In vitro* anticancer activity of extracts of *A. muricata* leaves

Extensive anticancer investigations have been conducted on *A. muricata* due to its reported ethnomedicinal uses against tumors and cancer (Adewole and Ojewole, 2009). Numerous lines of evidence suggest that the leaf extract of *A. muricata* repressed tumor growth *in vivo* in animal models as well as induced apoptosis of various cancer cells *in vitro* (Liu et al., 2016). Cytotoxicity studies on the *A. muricata* leaves have been accomplished on numerous cell lines. The *A. muricata* leaf extracts were investigated for cytotoxicity against human bladder cancer cells, K562 as well as human leukemia cancer cells, ECV304, where the results manifested a remarkable cytotoxic effects (Oviedo et al., 2009). The extract was also tested on kidney epithelial cells, VERO, stomach cancer cells, C-678 and human large lung cell carcinoma, H-460 cell lines and gave IC_50_ values lower than 0.00022 mg/mL for all three cell lines (Quispe et al., 2006). Subsequently, a number of cytotoxicity studies on the *A. muricata* leaf extract have been carried out, including cytotoxicity on histiocytic lymphoma cell line, U937, pancreatic cancer cells, FG/COLO357, breast cancer cells, MDA-MB-435S, immortalized human keratinocytes, HaCat, normal human liver cells, WRL-68 as well as human skin malignant melanoma, A375 (Ménan et al., 2006; Osorio et al., 2007; George et al., 2012; Nawwar et al., 2012; Torres et al., 2012). For the histiocytic lymphoma cell line, it was noted that the extract demonstrated considerable suppression with a LC_50_ value of 7.8 μg/mL. Moreover, it also demonstrated strong toxicity toward FG/COLO357 with an IC_50_ value of 200 μg/mL (Torres et al., 2012). George et al. (2012) reported the cytotoxic effect of n-butanolic extract of *A. muricata* leaves against MDA-MB-435S, HaCat and WRL-68 cell lines with IC_50_ values of 29.2, 30.1, and 52.4 μg/mL respectively.

Gavamukulya et al. (2014) investigated the cytotoxicity of the ethanol and aqueous extracts of *A. muricata* leaves on EACC, MDA, and SKBR3 tumor cell lines. The ethanol extracts showed IC_50_ values of 335.85, 248.77, and 202.33 μg/mL for EACC, MDA and SKBR3 tumor cell lines, respectively, and it showed no cytotoxic effect on normal spleen cells. The investigation also reported that the aqueous extract did not show any anticancer effect at all tested concentrations. Other than leaves, fruit, stems, and seeds of the *A. muricata* plant have also been reported to possess significant anticancer activities. In an *in vitro* study conducted on the fruit of *A. muricata* there was substantial repression of breast cancer cells growth (MDA-MB-468) as well as the selective suppression of the epidermal growth factor receptor (EGFR) (which regularly overexpressed in breast cancer) with IC_50_ of 4.8 μg/mL, while on non-tumorigenic human breast epithelial cells (MCF-10A) there was no effect (Dai et al., 2011). This promising investigation continued on mouse xenograft model and the results showed remarkable 56, 54, and 32.5% inhibition in protein expression of EGFR, p-EGFR, and p-ERK in MDA-MB-468 tumors, respectively, after 5 weeks of the administration of the *A. muricata* fruit extract (Dai et al., 2011). Recently, Sun et al. (2014) isolated and identified three novel annonaceous acetogenins, namely, muricin J, muricin K, and muricin L from the *A. muricata* fruit via chromatographic techniques and HPLC purification. These isolates were found to exhibit antiproliferative activity against human prostate cancer PC-3 cells. Apart from the leaves and fruits of the *A. muricata*, the stem also showed cytotoxic effect against various cell lines including U937, histiocytic lymphoma cell lines with IC_50_ of 10.5, 18.2, and 60.9 μg/ml exerted by the ethyl acetate, hexane and methanol extracts, respectively (Valencia et al., 2011). The stems also demonstrated cytotoxic activity by suppressing the expression of molecules associated to hypoxia and glycolysis in CD18/HPAF, pancreatic cancer cells (IC_50_:73.0 μg/mL) (Torres et al., 2012). Other than these, the ethanol extract of the seeds of *A. muricata* was also found to show cytotoxic effect on MDBK and HEp-2 cells (IC_50_ values: 34.5 and 55 mg/mL, respectively) at 24 h, and an IC_50_ value of 49.6 × 10^−3^ mg/mL toward HEp-2 cells at 72 h (Betancur-Galvis et al., 1999).

The hexane extract of the *A. muricata* leaves concentration-dependently inhibited the cell proliferation in pancreatic cancer cells (Capan-1) (Rosdi et al., 2015). Nineteen samples of *A. muricata* from different locations were screened against breast cancer cell lines; MCF-7, MDA-MB-231, and 4 T1. The results revealed that the aqueous extract of *A. muricata* leaves collected from Selangor, Malaysia showed the most potent effect with the lowest IC_50_ values of 220, 350 and 250 μg/mL for MCF7, MDA-MB231 and 4 T1 cell lines, respectively (Najmuddin et al., 2016). The extract also significantly decreased the size and weight of tumor, exhibited anti-metastatic properties, prompted apoptosis *in vitro* and *in vivo* of the 4 T1 cells as well as supressed the level of NO and malondialdehyde (MDA) in tumor while at the same time stimulated the level of white blood cell, T-cell, and natural killer cell population. Another study revealed that the chloroform, *n-*hexane and ethyl acetate extracts of the leaf of *A. muricata* possessed cytotoxic effect on Raji cells with IC_50_ values ranging from 90.6, 407.2 and 260.2 μg/mL. While, the cytotoxic effect of chloroform and *n*-hexane extracts on Hela cell gave IC_50_ values of 127.3 and 169.2 μg/mL, respectively (Artanti et al., 2016). Therefore it was confirmed from the aforesaid studies that the chloroform extract of the *A. muricata* leaves possessed potent cytotoxicity activity against Raji and Hela cells.

### *In vivo* anticancer activity of extracts of *A. muricata* leaves

Recently, it was reported that by modulating antioxidant enzymes in ICR male mice, the leaf extract of *A. muricata* caused blockage of 7, 12-dimethylbenz[a]anthracene (DMBA)/12-0- tetradecaboylphorbol-13-acetate (TPA)-induced skin tumorigenesis (Roduan et al., 2017). It was also explored that the hexane and dichloromethane extracts of the leaves of *A. muricata* incomparably blocked the tumor incidence as well as the tumor volume, while the methanol extract of the leaves displayed suppressive effects in comparison to carcinogen control group. In DMBA/TPA-induced mice, the administration of the leaf extract reduced the catalase, superoxide dismutase and lipid peroxidation levels to the normal levels (Roduan et al., 2017). Contrary to this, in DMBA-induced cell proliferation in the breast tissues of female albino mice, the ethanol extract of the *A. muricata* leaves exhibited chemo protective effect (Minari and Okeke, 2014). In addition, in rats, *A. muricata* leaves also demonstrated chemopreventive potential against azoxymethane-induced colonic aberrant crypt foci. (Moghadamtousi et al., 2015c). Another investigation reported that in liver cancer HepG2 cells, the ethanol extract of *A. muricata* leaves induced apoptosis through endoplasmic reticulum stress pathway (Liu et al., 2016). The proteomic analysis discovered 14 proteins associated with the extract in triggering apoptosis; including the up-regulation in HSP70, GRP94, and DPI-related protein 5 expression levels, which further confirmed the employment of the endoplasmic reticulum stress pathway by the extract. This investigation evidenced the potential of the *A. muricata* leaves extract as an effective anticancer agent. Subsequently, *A. muricata* extract was found to suppress the proliferation of HL-60 cells by inducing morphology changes, G0/G1 phase cell detention, damage to cell viability and detriment of membrane mitochondrial potential (Pieme et al., 2014). This finding proved that *A. muricata* has a promising potential as a chemotherapeutic agent to cure cancer. From all these findings, not only the leaves, the whole *A. muricata* plant parts were proven to be a versatile anticancer agent. Table 5 refers the key outcomes from prospective *in vitro* and *in vivo* oncogenic investigations on the various extracts of *A. muricata* leaves.

**Table 5:** Anticancer activity of *A. muricata* leaf extract.

**Type of activities**	**Extract/fractions**	**Subject**	**Dose**	**Key findings**	**References**
Antiproliferative	Ethanol Hexane	HL-60 cells Capan-1 cells		Suppressed the proliferation of HL-60 cells with IC_50_ values varied from 6-49 μg/mL by the interruption of reactive oxygen species (ROS) generation, MMP and the G0/G1 cell arrest. IC_25_ values ranging from ~7.8-8 and ~0.9-1.0 μg/mL, respectively.	Pieme et al., 2014; Rosdi et al., 2015
Antitumor	Ethanol, water	EACC, MDA & SKBR3 cell lines	0–1,250 μg/mL	The ethanol extract showed IC_50_ values of 335.85, 248.77, 202.33 μg/mL for EACC, MDA and SKBR3 cell line, respectively, and showed no effect on normal spleen cells, while the water extract exhibited no anticancer effect.	Gavamukulya et al., 2014
	Hexane, dichloromethane, methanol	DMBA/TPA induced mice		All the extracts suppressed the DMBA/TPA-induced skin tumor.	Roduan et al., 2017
	Ethanol	DMBA/croton oil-induced mice skin papillomagenesis		Suppressed the tumor initiation and promotion.	Hamizah et al., 2012
	Ethanol, water	Tumors induced mouse (skin)	30 mg/kg	Inhibited the initiation and promotion of tumors induced in mouse skin.	Hamizah et al., 2012
Breast cancer	Ethanol Aqueous	Female albino rats strain Sprague Dawley MDA-MB231 and 4 T1 cell lines	200, 300, 400 & 500 mg/kg	Reduced the proliferative indexes of breast cancer-induced DMBA and showed the most significant reduction at 300 mg/kg. The extract gave lowest IC_50_ values of 220, 350 and 250 μg/mL for the mentioned cell lines, respectively.	Najmuddin et al., 2016; Sulistyoningrum et al., 2017
Colon cancer	Ethyl acetate	HT-29 and HCT-116 cells		Exhibited remarkable cytotoxic effects on HT-29 and HCT-116 cells with IC_50_ values of 11.43 ± 1.87 and 8.98 ± 1.24 μg/mL, respectively.	Moghadamtousi et al., 2015c
	Ethyl acetate	Rats	250 and 500 mg/kg	Down-regulated the Bcl-2 and PCNA proteins and up-regulated the Bax protein.	Moghadamtousi et al., 2015c
	Ethanol	Rats	100 mg/kg	The extract restored colon total protein in cycas-induced colorectal carcinogenesis in rats.	Paulinus et al., 2013
Colorectal cancer	96% Ethanol	COLO-205 cell line	10 μg/mL	Caspase-3 increased 1.09 times by the extract and showed remarkable caspase-3 activity compared to leucovorin and placebo in the COLO-205	Abdullah et al., 2017
Cytotoxic	40% Ethanol	K562 and ECV-304		Showed MIC values of 7 and 2 mg/mL for K562 and ECV-304 cell lines, respectively.	Oviedo et al., 2009
	Ethyl acetate	U937 cells		The extract showed a LC_50_ value of 7.8 μg/mL	Osorio et al., 2007
	Ethanol	VERO, C-678 and H460 cells		Showed IC_50_ values lower than 0.00022 mg/mL for all three cell lines	Quispe et al., 2006
	DMSO	PC FG/COLO357		The extract showed IC_50_ value of 200 μg/mL.	Torres et al., 2012
	Butanol	MDA-MB-435S, HaCat and WRL-68 cells		Showed IC_50_ values of 29.2, 30.1 and 52.4 μg/mL for MDA-MB-435S, HaCat and WRL-68 cell lines, respectively.	George et al., 2012
	Ethanol: water	HaCat		At 1.6–50 μg/mL of concentration, the extract increased the cellular activity of the cell, while at 100 μg/mL, the extract showed no effect to the cells.	Nawwar et al., 2012
	Water, ethanol, pentane Chloroform, *n-*hexane and ethyl acetate	A375 Raji and hela cell lines		The ethanol and pentane extracts showed IC_50_ values of 320 and 140 μg/mL, respectively, while the water extract exhibited an IC_50_ value higher than 500 μg/mL. The respective extracts possessed cytotoxic effect on Raji cells with IC_50_ values of 90.6, 407.2 and 260.2 μg/mL. While, the chloroform and *n*-hexane extracts showed cytotoxic effect on Hela cell with IC_50_ values of 127.3 and 169.2 μg/mL, respectively.	Ménan et al., 2006; Artanti et al., 2016
Liver cancer	Ethanol	HepG2 cells	0 to 240 μg/mL	The number of cells in the sub-G1 fraction increased by the extract in a dose-dependent manner. The extract stimulates HepG2 cell apoptosis through ROS pathway.	Yang et al., 2015
	Ethanol	HepG2 Cell	120 μg/mL	Up-regulated HSP70 and GRP94 protein levels, as well as the phosphorylation of p-ERK and elF2α, and the expression level of Bip and CHOP. The extract also reported to induce ER stress.	Liu et al., 2016
	Water	Huh-7 cell	0.5 to 1.5 mg/mL	Showed antiproliferative activity and cytotoxic effects toward Huh-7 cell, possibly through the induction of apoptosis.	Banerjee et al., 2017
Leukemia	Ethanol	K562 cells		Induced the apoptosis of the cells.	Ezirim et al., 2013
Lung cancer	Ethyl acetate	A549 cells		Exhibited mitochondrial-mediated apoptosis and cell cycle arrest at G_1_ phase.	Moghadamtousi et al., 2014
Prostate cancer	Water	Rats	30 and 300 mg/mL	Reduced the size of the rats prostates after 2 months of treatment.	Asare et al., 2015

### Anticancer activity of the bioactive isolates of *A. muricata* leaves

**Figure 2:**
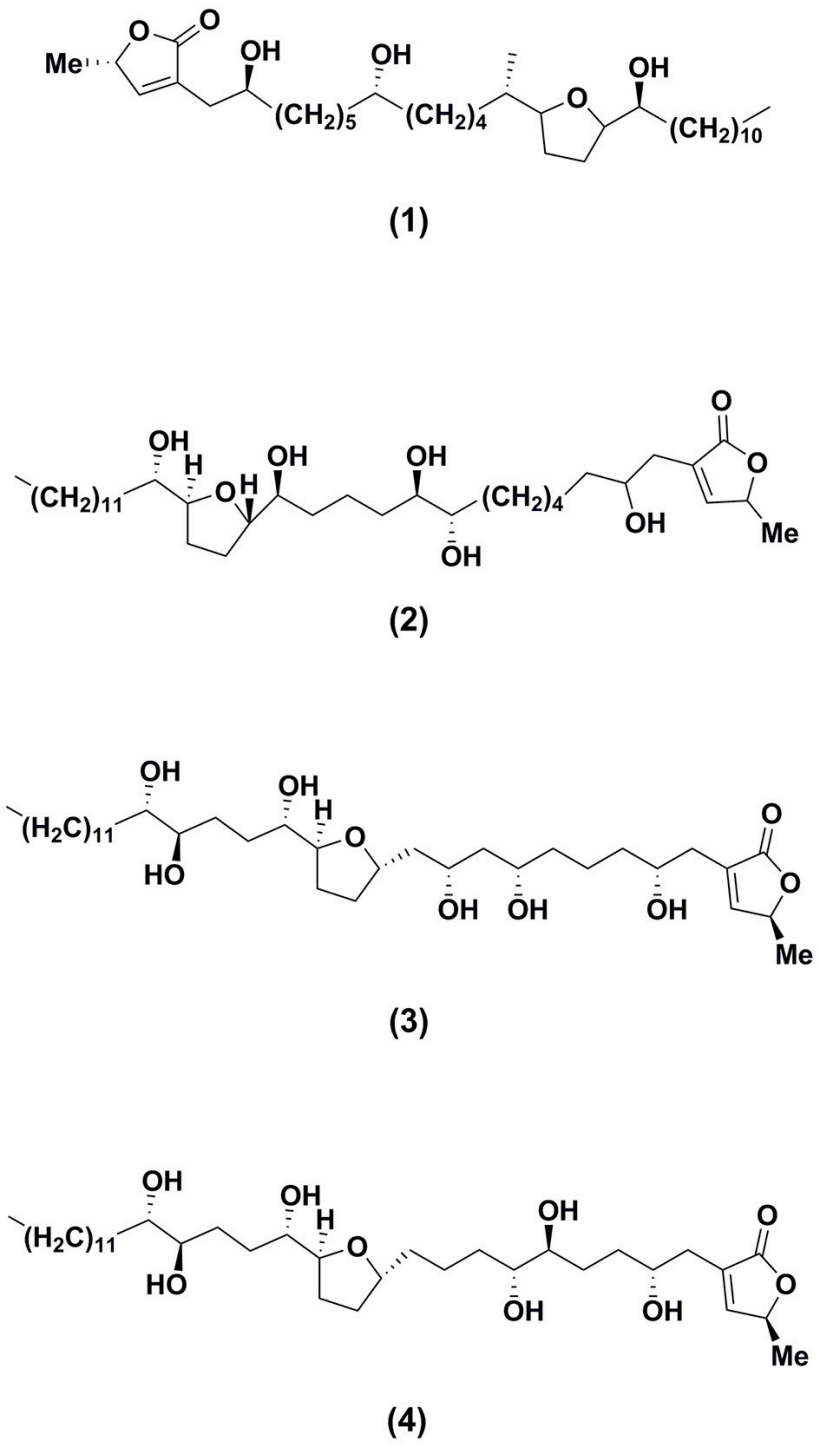
Structures of major bioactive isolates of *Annona muricata* leaves responsible for anticancer effects.

Besides the findings generated from the crude extracts, further investigations have been conducted on the isolated bioactive compounds from the leaves of *A. muricata*, in order to have a detailed understanding of the mechanistic effects of the respective biological properties. However, only a few of the isolates have been investigated on their biological and pharmacological activities especially anti-inflammatory and anticancer activities. Table 6 lists the anticancer activity of the major bioactive isolates of *A. muricata* leaves. Annonaceous acetogenins, alkaloids, and phenols are the bioactive metabolites isolated from the *A. muricata* leaves, and among them, annonaceous acetogenins are the most predominant one. The annonaceous acetogenins are a class of natural products that distinctively belong to Annonaceae family. These bioactive compounds exhibited an array of bioactivities such as immunomodulatory, anti-inflammatory, anticancer, antiparasitic, insecticidal, antimicrobial, neurotoxic, antileishmaniasis, and antioxidant. The leaves, as well as the stems of *A. muricata*, exhibited active cytotoxicity against cancer cells, owing to these acetogenins, which did not show toxicity toward normal cells, but highly toxic to cancerous cells (Oberlies et al., 1995; Villo, 2008). Annonacin is the ubiquitous acetogenin present in *A. muricata* leaves (Yuan et al., 2003; Champy et al., 2004). The chemical structures of annonaceous acetogenins including annonacin and annomuricin E, isolated from the *A. muricata* leaves are shown in Figure 2.

**Table 6:** Anticancer activity of the major bioactive isolates of *A. muricata* leaves.

**Bioactive isolates**	**Subject**	**Dose**	**Key findings**	**References**
Annonacin	T24 cells		Induced the cell cycle arrest in T24 bladder cancer cells and caused cytotoxicity in a Bax and caspase-3-related pathway	Yuan et al., 2003
	DMBA/TPA induced skin tumorigenesis mice MCF-7 cells	0.1 μM	Exhibited strong antitumor activity Annonacin induced growth arrest and apoptosis in ER-related pathways in MCF-7 cells	Ko et al., 2011; Roduan et al., 2017
Annomuricin E	HT-29 cells	4 to 16 μg/mL	Showed IC_50_ values of 5.72 ± 0.41, 3.49 ± 0.22 and 1.62 ± 0.24 μg/mL after 12, 24 and 48 h of treatments. Induced LDH leakage in cells at 1 and 2 μg/mL and exhibited significant LDH release at the concentration from 4 to 16 μg/mL. Induced the cell cycle arrest at G1, the phosphatidylserine externalization, the caspase activation, the mitochondria-initiated events, as well as Bax up-regulation and Bcl-2 down-regulation.	Moghadamtousi et al., 2015c
Muricoreacin	PC-3 cells		Selectively cytotoxic against the PC-3 cell line with five times higher activity compared to the positive antitumor control adriamycin.	Kim et al., 1998b
Murihexocin C	PACA-2 cells PC-3 cells		Showed selective cytotoxicity against the PACA-2 as well as the PC-3 cell lines.	Kim et al., 1998b

### Annonacin

Yuan et al. (2003) reported annonacin **(1)**, a mono-tetrahydrofuran acetogenin isolated from the seeds of *A. reticulata*, induced the cell cycle detention at the G1 phase in T24 bladder cancer cells by causing stimulation of p21, and also caused cytotoxicity in a Bax and caspase-3-related pathway. Subsequently, on 7, 12-dimethylbenz[a]anthracene (DMBA) induced and 12-0- tetradecaboylphorbol-13-acetate (TPA) promoted skin tumorigenesis in mice, the antitumor properties of different fractions of *A. muricata* leaves particularly hexane, dichloromethane, and methanol crude extracts were examined (Roduan et al., 2017). The outcomes of the experiment revealed the complete suppression of tumor formation in mice cured with the hexane and dichloromethane extracts of the leaves, respectively. While, the methanol extract showed significant delay in the tumor latency and attenuated the tumor incidence, tumor volume and tumor burden. Further investigation suggested that the hexane and dichloromethane extracts of the *A. muricata* leaves have higher content of annonacin. Therefore, the strong antitumor activity exerted by the extracts might be associated with the presence of cytotoxic constituents in the extracts particularly annonacin. The annonacin displayed its toxic effects on the cell by the suppression of mitochondrial complex I which resulted in the ATP depletion (Zafra-Polo et al., 1998; Yuan et al., 2003) and the repression of ubiquinone-linked NADH oxidase that is vital expression in cancer cells membrane which will kill the cancer cell and arrest the proliferation of cells (Woo et al., 1999). Besides, based on the study carried out by Ko et al. (2011), 0.1 μM of annonacin potentially induced growth arrest and apoptosis in ER-related pathways against MCF-7 cells.

### Annomuricin E

The effects of the annomuricin E **(2)**, an annonaceous acetogenin isolated from the ethyl acetate leaf extract of *A.muricata* on the HT-29 colon carcinoma cell lines and CCD841 normal colon cells were evaluated (Moghadamtousi et al., 2015c). Annomuricin E showed IC_50_ values of 5.72, 3.49, and 1.62 μg/mL for HT-29 cells, at time intervals of 12, 24, and 48 h of administrations, respectively. The results were found corresponding to the inhibitory potential exhibited by the standard anticancer drug used in the study, 5-FU. Annomuricin E showed a relatively high IC_50_ value for CCD841 with 32.51 μg/mL for 48 h, which demonstrated the lesser cytotoxic activities toward the normal cells. In addition, annomuricin E also prompted LDH leakage in HT-29 cells. The study reported that the annomuricin E increased the LDH leakage at concentration of 1 and 2 μg/mL and exhibited significant LDH release at the concentration from 4 to 16 μg/mL, parallel to the control cells, which were treated with 0.1% DMSO and displayed a low level of LDH after 24 h of treatment. The HT-29 cells showed significant LDH leakage at a dose of 4 μg/mL of annomuricin E. Annomuricin E, again proving its effectiveness as anticancer agent by inducing the cell cycle arrest at G1, the phosphatidylserine externalization (biochemical characterizations of apoptosis), the caspase activation, the mitochondria-initiated events, as well as Bax augmentation and Bcl-2 inhibition. All the data and parameters from the investigation conclude that annomuricin E inhibited the HT-29 cells proliferation selectively as well as prompted apoptosis, which is related to G1 cell cycle arrest and mitochondria-mediated signaling pathways.

### Muricoreacin and murihexocin C

Kim et al. (1998b) reported that both muricoreacin as well as murihexocin which were isolated from the 95% ethanol leaf extract *A. muricata* revealed substantial cytotoxic activities among six human tumor cells. Muricoreacin **(3)** was found particularly cytotoxic in contrast to the prostate adenocarcinoma (PC-3) cell line with 5 times higher activity compared to the positive antitumor control used, adriamycin. While, murihexocin C **(4)** showed selective cytotoxicity against the pancreatic carcinoma (PACA-2) as well as the prostate adenocarcinoma (PC-3) cell lines, with lower activity.

## Other biological activities of *Annona muricata* leaves

Besides the anti-inflammatory and anticancer properties, the leaves of *A. muricata* have also been thoroughly investigated for other pharmacological and biological properties. Bento et al. revealed that the *A. muricata* leaves showed significant antiulcer activity against lesions (Bento et al., 2016). Besides that, a remarkable wound healing properties by the ethyl acetate leaf extract of *A. muricata* has been discovered by Moghadamtousi et al. (2015b). In another investigation, the *A. muricata* leaves were showed to possess enzymatic antioxidants; superoxide dismutase and catalase, together with the non-enzymatic antioxidants for instance vitamin C and vitamin E (Vijayameena et al., 2013). The DPPH test revealed the significant antioxidant activity of the aqueous and ethanol extracts of *A. muricata* leaves (Gavamukulya et al., 2014). In addition, *A. muricata* leaves were also found to exhibit anti-plasmodial, anti-arthritic, anti-protozoal, antibacterial, antimicrobial, anticonvulsant, antidiabetic and hypolipidemic, antihypertensive, antiparasitic, insecticidal, gastroprotective, molluscicidal, hepatoprotective, and bilirubin-lowering activities (Moghadamtousi et al., 2015a).

## Clinical studies

At present, clinical trials on the standardized extract of *A. muricata* and its bioactive compounds may not be possible due to insufficient data generated from preclinical testings which include pharmacokinetic and toxicological studies. Despite the regulatory requirements for clinical studies, there were already two case studies carried out to evaluate the efficacy of *A. muricata* as an anticancer agent in human (Yap, 2013; Hansra et al., 2014). The first case involved a colon cancer patient who was subjected to combined lifestyle modifications with the intake of some herbal extracts and nutraceuticals, including a daily ingestion of 5 g of powdered leaf and seed of *A. muricata* extract. There was disappearance of the malignancy with substantial regression of colon tumor cells in the patient (Yap, 2013). Another study involved a breast cancer patient who was given 227 g of leaves decoction of *A. muricata* (10–12 dry leaves in water for 5–7 min) daily and capecitabina (2,500 mg PO) 2 weeks on 1 week off (Hansra et al., 2014). Tumor markers in the patient has been stable and had no side effects after therapy for 5 years. Since these are preliminary clinical trials with small sample size, more operationally thorough and serious randomized controlled trials have to be carried out to evaluate the plant as an effective and safe anti-inflammatory and anticancer agent for human consumption.

## Toxicology investigations

There are few data available on toxicity of *A. muricata* leaves and the data available are only on neurotoxicity and acute toxicity.

### Neurotoxicity studies

An epidemiological investigation was published by the Lancet Journal on possible relationship between atypical parkinsonism in the French West Indies with consumption of tropical fruits including *A. muricata* (Capparros-Lefebvre et al., 1999). The study postulated a link with consumption of herbal tea and fruits from the Annonaceae family (*A. muricata* and *A. squamosa*), which contain neurotoxic benzyltetrahydro-isoquinoline alkaloids, and Parkinson's disease. They suggested that chronic exposure to neurotoxic alkaloids could be an important aetiological factor because these compounds induce parkinsonism in animals. However, since this was a case-study involving small sample number (87 patients), a larger epidemiological study need to be carried out to clarify the link between these fruits with atypical parkinsonism and supranuclear palsy. This relationship has also been observed among New Caledonia and Caribbean patients living in London (Shaw and Hoglinger, 2008). Another study revealed that the ethiology of a neurodegenerative disease in Guadeloupe Island has a close correlation with consumption of annonaceous acetogenin. Annonacin present as the major compound depleted the ATP supply in rat striatal neurons and interrupted the transportation of mitochondria to the cell soma, which caused cellular pertubations in the protein tau and led to a number of similar characteristics as neurodegenerative diseases (Escobar-Khondiker et al., 2007). Bonneau et al. (2012) reported that annonaceous acetogenins were environmental neurotoxins responsible for Guadeloupean atypical Parkinsonism. The fruit of *A. muricata* was shown to contain high concentration of these compounds and its consumption constitute as a major source of exposure to these compounds and potential risk factors for neurodegeneration.

Quantification of acetogenins in *A. muricata* extracts by reversed phase HPLC and matrix-assisted laser desorption-ionization mass spectrometry (MALDI MS) showed an average *A. muricata* fruit contained about 15 mg of annonacin, a can of commercial nectar contained 36 mg, and a cup of infusion or decoction contained 140 μg (Champy et al., 2005). The neurotoxic effect of annonacin was determined by intravenous administration of annonacin (3.8 and 7.6 mg per kg per day for 28 days) to rats (Champy et al., 2004). Annonacin entered the brain parenchyma and decreased brain ATP levels by 44%, causing neuropathological abnormalities in the basal ganglia and brainstem nuclei. There was a significant loss of dopaminergic neurones in the substantia nigra, and cholinergic and dopamine and cyclic AMP-regulated phosphoprotein (DARPP-32)-immunoreactive GABAergic neurones in the striatum, accompanied by a significant increase in the number of astrocytes and microglial cells, based on stereological cell counts. The authors suggested that the distribution of the lesions was similar to that in patients with atypical parkinsonism. They also estimated that the amount of annonacin ingested in a year by eating one fruit or can of nectar a day were comparable to the dose of 3.8 mg/kg/day that induced widespread neurodegeneration when administered intravenously into rats for 28 days (106 mg/kg). However, without support of any bioavailability data on annonacin after intravenous or oral administration, this comparison was a crude estimation. Thus on the basis of available experimental data and the absence of detailed pharmacokinetic and pharmacodynamic data, preclinical and clinical studies, no confirmative conclusion on the link between consumption of *A. muricata* and atypical parkinsonism could be established yet. In 2010, l'Agence Francaise de Securite des Aliments (AVIS) issued a statement that it is not possible to conclude based on available experimental data that cases of atypical parkinsonism observed in Guadeloupe are linked to consumption of species belonging to Annonaceae family. However, it is advisable to avoid excessive and long term consumption of products containing *A. muricata* to prevent possible neurotoxicity.

### Acute toxicity studies

de Sousa et al. (2010) reported that the ethanol extracts of *A. muricata* leaves exhibited LD_50_ value of 1.67 g/kg at the dosages of 0.5, 1, 1.5, 2, and 3 g/kg. While Larbie et al. (2011) reported that the intake of the aqueous extract exceeds 5 g/kg might caused kidney problem, however, 1 g/kg consumption of the extract exhibited hypoglycemic and hyperlipidemia effects. The previous investigations revealed that the methanol extracts of *A. muricata* pericarp, fruit pulp as well as the seeds were most toxic among the other parts of the plant (Boyom et al., 2011). On the other hand, Yang et al. (2015) have investigated the toxicity of *A. muricata* leaf extract together with its flavonoid- and acetogenins-enriched extracts and they revealed that the acetogenins-enriched extract was more toxic than the other extracts. Subsequently, Quilez et al. (2015) reported an acute toxicity study on the *A. muricata* leaves. The experiment was conducted using 5 week old Swiss albino male mice which were assigned randomly into a control group and three dosages groups with doses of 250–1,000 mg/kg. The animals were monitored 3 h after the treatment of the extract to observe any indication of toxicity or mortality, and then, for the next 48 h. The single dose of 250, 500, and 1,000 mg/kg did not show any behavioral changes in the treated animals and no mortality was reported during the study. The macroscopic pathology interpretations showed that there was no visible lesion in any treated animals. From the finding, it was confirmed that the *A. muricata* leaf extract did not show toxicity in the murine peritoneal macrophages by assessing with mitochondrial reduction of MTT (Quilez et al., 2015).

## Conclusion & future prospects

Conclusively, *A. muricata* leaf and its secondary metabolites produce anti-inflammatory, anti-cancer and other immune system related effects. Literature reviews have identified and reported 117 isolates from the leaf of *A. muricata*, consisting mainly of annonaceous acetogenins, alkaloids, and phenolic compounds. In this review, we summarized versatile pharmacological activities particularly anti-inflammatory and anti-cancer effects possessed by aforesaid species. Still, there is much research gap and eventual studies need to be conducted for detailed investigations and better understanding of anti-inflammatory and anticancer potential exerted by *A. muricata*. In addition, most of the biological and pharmacological studies conducted using the crude extracts of the species are at its preliminary stages. The bioactive compounds contributing to the bioactivities have not been properly identified, qualitatively and quantitatively analyzed as chemical markers for standardization and quality control purposes as well as the mechanisms of action have not been well determined. Hence, future research on *A. muricata* should focus on extensive phytochemical investigations in isolating and identifying the active metabolites which contribute to the potent anti-inflammatory and anti-cancer activities. Subsequently, the extract and their active metabolites should also be subjected to more mechanistic studies, *in vivo* investigations in various animal models including pharmacokinetic and bioavailability studies. In addition, more toxicity studies must be conducted before submission to clinical trials to define the safest concentration of the *A. muricata* leaf to subjects. The potential of *A. muricata* leaves to be used as an anti-inflammatory and anticancer agent can clearly be enlightened by understanding its mechanisms of action on the human body system.

## Author contributions

SMAW drafted the manuscript. IJ participated in the concept, editing, and the final approval of the final version of the manuscript to be submitted for publication. MAH and LA were involved in the editing process.

### Conflict of interest statement

The authors declare that the research was conducted in the absence of any commercial or financial relationships that could be construed as a potential conflict of interest.

## Abbreviations

ACF: colonic aberrant crypt foci
*A. muricata*: *Annona muricata* L.
ATP: adenosine triphosphate
DMBA, 2: 4-dimethoxybenzaldehyde
DPPH, 2: 2-diphenyl-1-picrylhydrazyl
ECM: extracellular matrix
EGFR: epidermal growth factor receptor
EGFR: epidermal growth factor receptor
ER: endoplasmic reticulum
ERK: extracellular signal-regulated kinases
5-FU: fluorouracil
HPLC: high-performance liquid chromatography
HEp-2: human epithelial type 2 cells
Hsp70: 70 kilodalton heat shock protein
IL-1β: interleukin-1 beta
IL-6: interleukin-6
LPS: lipopolysaccharides
LDH: lactate dehydrogenase
MDBK: morphological observation of bovine kidney
MMPs: matrix metalloproteinases
MPO: myeloperoxidase
MTT, 3-(4, 5-dimethylthiazol-2-YI)-2: 5-diphenyltetrazolium bromide
NADH: nicotinamide adenine dinucleotide(NAD)+hydrogen(H)
NO: nitric oxide
OECD: Organization for Economic Co-operation and Development
ROS: reactive oxygen species
TPA: tetradecanoylphorbol acetate
TNF-α: tumor necrosis factor-alpha
VCAM-1: vascular cell-adhesion molecule-1.
